# Berberine augments the secretory function of salivary gland in homeostasis and after radiation exposure

**DOI:** 10.3389/fimmu.2025.1685137

**Published:** 2026-01-12

**Authors:** Qihang Lian, Yue Tian, Nan Wang, Yikun Luo, Xi Wang, Banghui Liu, Hefei Tian, Xiangjun Liu, Qingping Yin, Zhenni Xu, Yujun Huang, Lingxiao Huang, Xudan Lei, Jinyi Lang, Mei Feng, Dengqun Liu

**Affiliations:** 1 Precision Radiation in Oncology Key Laboratory of Sichuan Province, Department of Experimental Research, Sichuan Cancer Hospital & Institute, Sichuan Provincial Engineering Research Center of Tumor Organoids and Clinical Transformation, Sichuan Clinical Research Center for Cancer, Sichuan Cancer Center, University of Electronic Science and Technology of China, Chengdu, China; 2 Department of Medical Oncology, The Third People’s Hospital of Sichuan Province, Chengdu, China

**Keywords:** berberine, homeostasis, inflammation, proliferation, radiation, submandibular gland

## Abstract

**Introduction:**

Radiotherapy serves as an essential therapeutic modality for head and neck malignancies. However, many patients who undergo head and neck radiation (HNR) frequently experience different severities of xerostomia. Berberine (BBR) has a variety of pharmacological functions and has shown favorable clinical efficacy. However, its therapeutic potential and mechanistic basis in xerostomia have not been explored.

**Methods:**

The histological expressions of Aquaporin 5 (AQP5), Na-K-Cl cotransporter 1 (NKCC1), Muscle intestine stomach expression 1 (MIST1), Proliferating cell nuclear antigen (PCNA), Phospho-GSK-3beta (p-GSK3β) and β-Catenin were examined by immunohistochemistry (IHC). Mucin2 (MUC2), were examined by immunofluorescence. The degree of apoptosis was assessed by TUNEL. The mRNA expression levels of AQP5, NKCC1, PCNA, MUC2, and MIST1 were detected by qRT-PCR assay. The degree of inflammatory was evaluated by detecting the mRNA expression levels of *Il1b*, *Tgfb1*, *Tnf*, and *Il10*. The Proliferation level was performed by salivary gland organoids.

**Results:**

BBR significantly enhanced saliva secretion in normal physiological conditions and after radiation injury. Mechanistically, BBR upregulated the expression of AQP5, NKCC1 and MIST1. Moreover, BBR conferred its protection via the upregulation of mucin 2 (MUC2) expression, and qPCR analysis revealed elevated *Bhlha15* levels. Additionally, BBR preserved cellular proliferation, decreased TUNEL^+^ apoptotic cells and the inflammatory response in SMG tissues and organoids in HNR-induced xerostomia models.

**Conclusion:**

In conclusion, this study demonstrates that BBR can increase saliva secretion in healthy and HNR mice, indicating its potentiality for the treatment of radiation-induced xerostomia.

## 1 Introduction

Xerostomia is a common clinical symptom characterized by persistent subjective oral dryness due to the dysfunction of the salivary glands, especially for patients with malignancy after head and neck radiotherapy (HNR) (1). However, there is still a lack of effective countermeasures for xerostomia. In mammals, the salivary glands usually include the parotid gland (PG), SMG, and sublingual gland (SG). Among these glands, the SMG is the major salivary gland in mice. Histologically, the SMG comprises two principal cellular components: serous acinar cells and mucous ductal cells, which are responsible for primary saliva production and ion transportation, respectively. Notably, ductal cells exhibit minimal secretory capacity under physiological conditions (2). Radiation-induced xerostomia primarily arises from the proliferation suppression and concurrent apoptosis potentiation of SMG cells after radiation exposure (3), and such a pathological program is further exacerbated by localized inflammatory responses and decreased expression of functional genes, such as Aqp5, Nkcc1 and Mist1, which are necessary for the maintenance of normal saliva secretion (4, 5). Together, these two aspects lead to impaired secretory function and the depletion of acinar cells in the salivary glands (6).

The dysfunction of saliva secretion also subsequently has many harmful impacts on oral health. For example, diminished saliva secretion manifests clinically as increased susceptibility to dental demineralization, compromised antimicrobial defenses against oral pathogens, and significant deterioration in the quality of life of patients (7, 8). The current therapeutic strategies for xerostomia, including interventional therapy bundles (9), low-level light therapy (10), and allogeneic mesenchymal stem cell transplantation (11), have exhibited varying degrees of clinical efficacy. Deciphering the biological and molecular changes in salivary gland is the premise for more effective therapeutics of xerostomia. Transcriptomic profiling has revealed key molecular regulators of salivary gland dysfunction (12), thus providing insights into potential therapeutic targets and paving the way for future clinical benefits in patients. Nevertheless, a considerable distance remains between these mechanistic discoveries and their clinical application, especially the high development costs. The absence of standardized therapeutic protocols still persists owing to cost constraints and limited technical accessibility. Therefore, new drugs and intervention protocols for the potential clinical management of xerostomia are urgently needed.

Berberine (BBR) is an isoquinoline alkaloid, which is the major component of the natural extract of Coptis (13). BBR has multiple pharmacological functions. For example, it could induce cell cycle arrest, suppress cancer metastasis, and sensitize chemotherapy (14), and it is also a comprehensive metabolic regulator (15). BBR is also used to treat of neuropsychiatric disorders (16, 17) and suppress the production of proinflammatory cytokines (18). Moreover, BBR exhibits protective effects in models of gastrointestinal inflammation (19). BBR could modulate apoptotic signaling pathways via the regulation of apoptosis associated gene expression (20). Our prior study has highlighted the therapeutic effects of BBR on radiation-induced intestinal injury (13). BBR is able to maintain the mucosal barrier function and regulate mucosal immune homeostasis through Wnt/β-Catenin pathway (21). These mechanisms are highly relevant to the health and radiation injury of salivary gland, which relies on the precise regulation of critical processes, such as tissue inflammation, apoptotic signaling, and mucosal immune homeostasis. All the above pharmacological properties provide us with a compelling rationale for exploring the biological effects of BBR on saliva secretion and its utility for treating xerostomia.

Given the high morbidity of xerostomia in patients with head and neck malignancies after radiotherapy, elucidating the connections between BBR and saliva secretions is important and might provide novel therapeutic targets for xerostomia management. However, few studies have directly investigated the therapeutic associations between BBR and xerostomia. Therefore, the cellular and molecular mechanisms responsible for the modulatory effects of BBR on saliva secretion, particularly in the context of radiation-induced xerostomia, remain poorly characterized. In this study, we determined how BBR affects saliva secretion in homeostasis in the absence of injury and in HNR-induced xerostomia murine models. And it was discovered that BBR enhanced the secretory function of the salivary gland in healthy and HNR-treated mice. These results suggest a novel possibility that BBR and its derivatives could be used for the prevention and intervention of patients with xerostomia after head and neck radiotherapy.

## 2 Materials and methods

### 2.1 Animals

Six- to eight-week-old adult C57BL/6 mice with a body weight of 18~22 g were purchased from HFK Bioscience Co., Ltd. (Beijing, China). When the mice arrived, they were acclimated for three days before experimental use. All the mice were housed in a specific pathogen-free (SPF) facility under a 12-hour light/dark cycle with free access to food and water. All the experimental procedures in this study were performed in compliance with the Guidelines for Care and Use of Laboratory Animals of NIH (8^th^ edition), and the experimental protocols were approved by the Ethics Committee of Sichuan Cancer Hospital and Institute (SCCHEC-04-2023-014).

### 2.2 Head and neck irradiation and BBR treatment

Adult mice were randomly assigned to different experimental groups to minimize subjective bias. The group assignments were as follows: the healthy control group, the BBR group, the head and neck radiation (HNR) group, and the HNR+BBR group. BBR chloride (HY-18258, MCE, China) was freshly dissolved in distilled water. For the homeostatic study, the mice were treated by intraperitoneal (i.p.) injection of BBR at a dosage of 5 mg/kg/day for 6 consecutive days. For the HNR experiment, the mice were first anesthetized with 1% sodium pentobarbital. The head and neck areas were irradiated with a single dose of 15 Gy via X-Rad 320 irradiation (PXI, Connecticut, USA), and the dose rate was 75 cGy/min. The local head and neck irradiation range was 1.5 cm above the flat upper limb, and other parts of the body were covered with a 1.5-cm-thick lead plate to protect the surrounding tissues. After irradiation, the mice in the HNR+BBR group were treated with an i.p. injection of BBR (5 mg/kg/day) throughout the entire experiment. The mice in the control group and HNR group were given the same volume of PBS at the same time. The preparation and administration of BBR were performed by different investigators to minimize the experimental bias.

### 2.3 General observations and salivary flow measurement

The mice in each group were observed, weighed, and documented daily throughout the study. Saliva secretion was assessed at the indicated time points (healthy group, 1 d, 3 d, and 7 d; HNR group, 1 d, 3 d, and 7 d). The measurement was performed as previously described (22). Briefly, before measurement, the mice were intraperitoneally injected with 0.1 g/kg pentobarbital sodium. When the mice were anesthetized, they were given an intraperitoneal injection of 0.5 mg/kg pilocarpine to stimulate saliva secretion for 5 min. Then, the mice were placed on their left side to collect saliva with cotton balls for 20 min. After collection, the cotton balls were weighed again to calculate the net saliva secretion.

### 2.4 Tissue collection and sample preparation

The mice were sacrificed by cervical dislocation at the indicated time points, and their SMG tissues were quickly removed and fixed in 4% precooled paraformaldehyde (PFA, #BL539A, Biosharp, China) for 3 days. After fixation, the SMG tissues were processed by regular dehydration and paraffin embedding following a standard histological protocol in the laboratory. Tissue blocks were prepared from SMG tissues, and paraffin sections were prepared at a thickness of 4 μm. The tissue sections were mounted onto microscope slides and then stained with conventional hematoxylin and eosin (H&E) with a commercial staining kit (C0105M, Beyotime, China).

### 2.5 Alcian blue staining

Because Alcian blue can be used to stain acidic mucus polysaccharides, we used it to stain tissue slides to determine the level and distribution of acidic mucin in SMG tissues. Briefly, tissue slides were deparaffinized and rehydrated by a standard protocol in the laboratory. The tissues were stained with a commercial kit for Alcian blue staining (#E670107, BBI) following the protocol provided by the manufacturer. Alcian blue solution was added to the tissues, which were then incubated for 30 min at room temperature. Afterward, the SMG tissues were washed in PBS for 5 min three times. The cell nuclei were stained with fast red or hematoxylin solution for 3 min. Finally, the tissue slides were dried and mounted with neutral resin for subsequent observation.

### 2.6 Immunostaining

SMG tissue sections were dehydrated with xylene and graded ethanol solutions following a conventional protocol. After rehydration, the tissue slides were boiled in Tris-EDTA Antigen Retrieval Solution (#BL618A, Biosharp, China) for 20 min to perform antigen retrieval. After the slides were cooled, they were washed and blocked with 1% bovine serum albumin (BSA, #A7906, Sigma Aldrich) supplemented with 0.5% Triton X-100. The primary antibodies were freshly diluted and then incubated with the tissues overnight at 4 °C. The diluted antibodies used in this study included those against AQP5 (1:400), NKCC1 (1:200), CK7 (1:400), MIST1 (1:250), PCNA (1:400), MUC2 (1:400), p-GSK-3β (1:200), and β-Catenin (1:200). Detailed information on the antibodies is listed in **Supplementary Table S1**. On the second day, the primary antibodies were carefully removed, and HRP-linked ready-to-use goat anti-rabbit or mouse serum (ZSBio, Beijing, China) was added and incubated for 30 min at room temperature. For immunohistochemistry (IHC)-based signal visualization, a DAB staining kit (ZSBio, Beijing, China) was used. The cell nuclei were counterstained with hematoxylin. For immunofluorescence staining, Alexa Fluor™ 488-conjugated highly cross-adsorbed donkey anti-rabbit IgG (H + L) antibodies (Thermo Fisher, USA) were used, and the nuclei were stained with DAPI (Vector Laboratories, Burlingame, CA). Finally, the slides were mounted with ProLong^TM^ Gold anti-fade reagent (Thermo Fisher, USA).

### 2.7 One-step TUNEL apoptosis assay

The apoptosis of submandibular gland cells after BBR or HNR treatment was examined by a One-Step TUNEL Apoptosis Kit (KeyGen Bio-Tech, Nanjing, China) following the manufacture’s protocol. Briefly, the slides were dewaxed in xylene and serial ethanol dilutions and rehydrated in distilled water. SMG tissues were incubated with Proteinase K at 37 ^°^C for 30 min to expose DNA, and then FITC-labelled TUNEL reaction solution was incubated at 37 ^°^C for 1 h in the dark chamber. After washing out the unbinding reaction buffer, the nuclei were stained with DAPI (Vector Laboratories, Burlingame, CA). Finally, the slides were also mounted with ProLong^TM^ Gold anti-fade reagent (Thermo Fisher, USA). Slides were observed using a Zeiss Axio Observer with Apotome3.

### 2.8 RNA preparation and qRT–PCR assay

During tissue collection, SMG tissue samples from each group were stored at -80°C after flash freezing in liquid nitrogen. Total RNA was extracted with RNAiso Plus (9109, Takara, Japan) following the manufacturer’s recommendations. The quality and concentration of the RNA samples were analyzed with a NanoDrop2000 spectrophotometer (Thermo Scientific, USA). Reverse transcription was performed to obtain cDNA using Hifair II 1^st^ Strand cDNA Synthesis SuperMix (11137ES60, YEASEN, China). qPCR was conducted with Hieff qPCR SYBR Green Master Mix (11203ES08, YEASEN, China) using a C1000 instrument (Bio-Rad, USA). Gene-specific primers for *Aqp5, Slc12a2, Bhlha15, Pcna, Il1b, Il10, Tfgb1, Tnf*, and *Actb* were synthesized by BGI (Chengdu, China). The sequences of primers used in this study are listed in **Supplementary Table S2**. Gene expression levels were normalized to that of Actb, and relative expression was determined by the 2*^−ddCt^* method.

### 2.9 Culture of salivary gland organoids

Salivary gland organoids (SGOs) were cultured following a previously described protocol (23). Briefly, fresh SMG were quickly removed from the mice and placed in cold PBS. The superficial fascia coating the SMG was carefully removed to expose SMG tissues. The slides were thoroughly washed three times in PBS containing 3% penicillin and streptomycin and then chopped into small tissue fragments. SMG tissue fragments were digested for 30 min in a 37 °C incubator in a 5% CO_2_ atmosphere. The digestive buffer was prepared with Dulbecco’s modified Eagle’s medium (DMEM) supplemented with 1 mg/mL collagenase I (Sigma–Aldrich, MO, USA). After digestion, the tissue fragments were centrifuged at 1000 rpm for 3 min. The cell pellets were resuspended in DMEM and filtered through a 40-μm nylon cell strainer (BD Biosciences, NJ, USA) to collect the single-cell suspensions. After centrifugation at 1200 rpm for 5 min, the glandular cells were resuspended in Matrigel (Corning, MA, USA) and seeded into a 96-well plate. The plate was incubated at 37 °C for 10 min to allow the Matrigel to solidify. Finally, complete SGO culture medium, which was prepared with Advanced DMEM/F12 (Gibco, USA) supplemented with 20 ng/mL EGF (PeproTech, USA), 20 ng/mL FGF-2 (PeproTech, USA), 1% N2 supplement (Thermo Fisher, USA), 1% insulin-transferrin-selenium (Thermo Fisher, USA), 1 μM dexamethasone (Sigma–Aldrich, USA), and 2% penicillin–streptomycin (Beyotime, China), was loaded into the wells. The culture medium was replaced every three days. Depending on the dose screening results, BBR (10 µM) were supplemented into the organoid culture medium independently or in the combination of either 80 mM LiCl (HY-Y0649, MCE, China) or 5 µM XAV939 (HY-15147, MCE, China). For the radiation experiments, SGOs were firstly cultured for 5 days and then irradiated by a single dose of 6 Gy, then BBR was loaded with or without XAV939.

### 2.10 Image capture and quantitative analysis

Images of the histologically stained tissues were captured with a BX53 bright field microscope (Olympus, Japan). Immunofluorescent histological images were obtained with an Axio Observer with Apotome3 (Zeiss, Germany). SGO images were acquired by M5000 (Thermo Fisher, USA) and Cytation 5 (BioTek, USA) microsystems. The parameters of all the images, such as the positive signal area and staining intensity, were analyzed by ImageJ software (NIH, USA). During the quantification analysis, researchers were blinded to the information of the specific images.

### 2.11 Statistical analysis

All the values are presented as the means ± SDs, and the data were analyzed with GraphPad Prism 9 (GraphPad Software, USA). Comparisons between two different groups were conducted by two-tailed unpaired Student’s *t* test. Comparisons between multiple groups were performed using one-way ANOVA with *post-hoc* Tukey’s test. Differences with *P* values less than 0.05 were considered statistically significant. *: *P* < 0.05, **: *P* < 0.01, ***: *P* < 0.001, ****: *P* < 0.0001. *P* > 0.05 was considered to indicate a nonsignificant (*n.s.*) difference.

## 3 Results

### 3.1 BBR promotes the saliva secretion from the SMG of healthy mice

**Figure 1:**
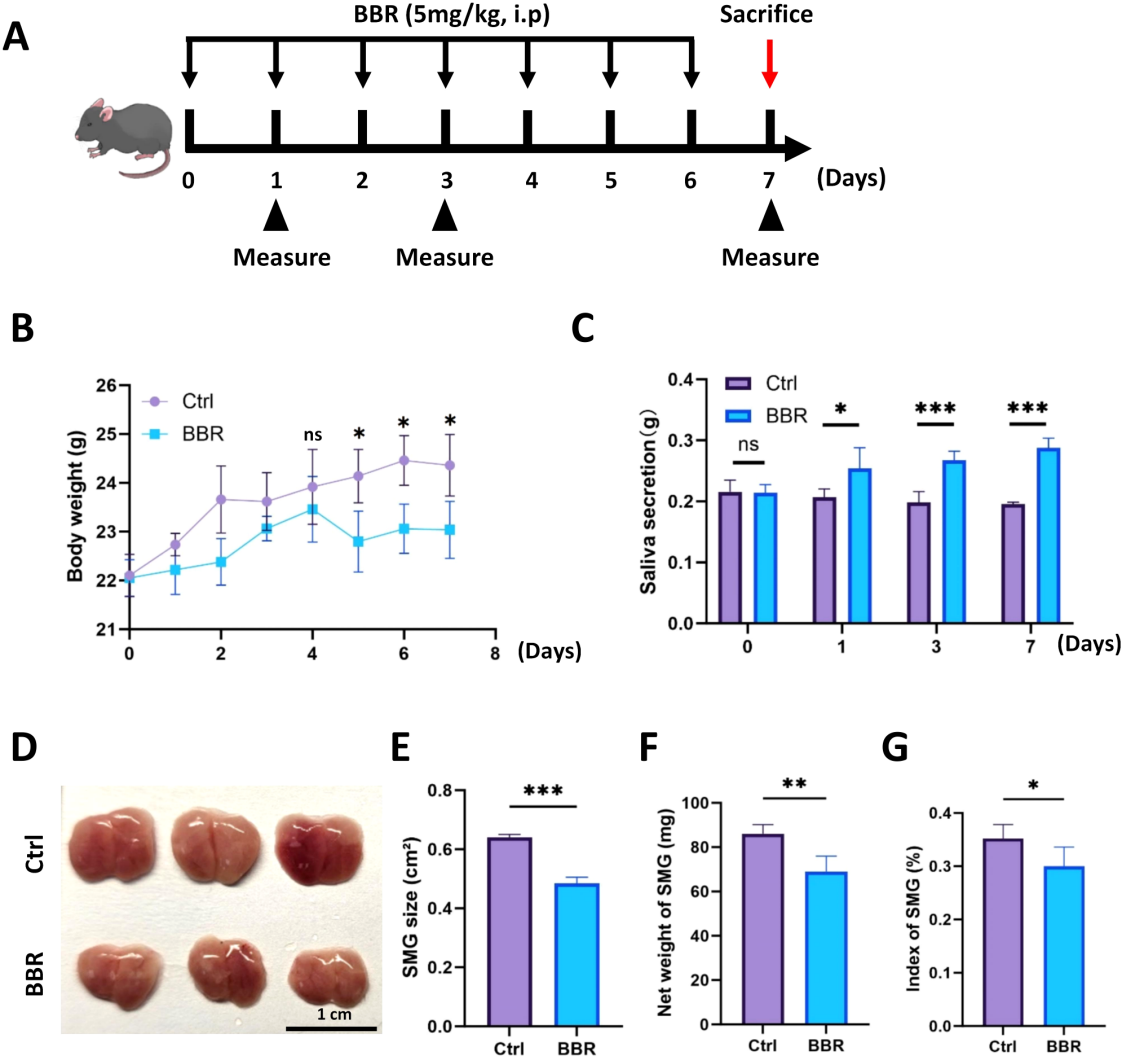
BBR increases saliva secretion under physiological conditions. **(A)** Schematic diagram showing the administration of BBR (5 mg/kg/day) to mice under physiological conditions. **(B)** BBR treatment inhibited the increase in body weight in healthy mice (n=4 each group). **(C)** Saliva secretion was significantly greater in BBR-treated mice than in untreated healthy mice (n=4 in each group). **(D)** Gross morphology of SMG in different groups (n=3 each group, Bar= 1 cm). **(E)** Analysis of SMG surface area in control and BBR-treated mice (n=4 per group). **(F)** Measurement of SMG net weight in control and BBR-treated mice (n=4 per group). **(G)** Normalized SMG index in control and BBR-treated mice (n=4 per group). *P < 0.05, **P < 0.01, ***P < 0.001, *ns*, not significant.

At the beginning of this study, we firstly evaluated whether BBR affects salivary secretory function under physiological conditions. We treated healthy mice with BBR (5 mg/kg) by intraperitoneal injection for 6 consecutive days, and the mice were sacrificed on day 7 to collect SMG tissues (**Figure 1A**). BBR-treated mice presented a lower increase in body weight (**Figure 1B**), which was consistent with our previous observations (13). Interestingly, we observed that the mice in the BBR-treated group had a significantly increased amount of saliva secretion (**Figure 1C**). However, when we collected the whole SMG of these two groups, the BBR-treated SMG had an obviously decreased size according to their gross appearance (**Figure 1D**). Moreover, the gross, net weight, and index of SMG normalized to their body weight showed that BBR-treated mice all had a significantly shrunk and smaller SMG (**Figures 1E–G**). Therefore, these results demonstrate that BBR is capable of increasing saliva secretion during homeostasis.

### 3.2 BBR changes the histological characteristics of the SMG

**Figure 2:**
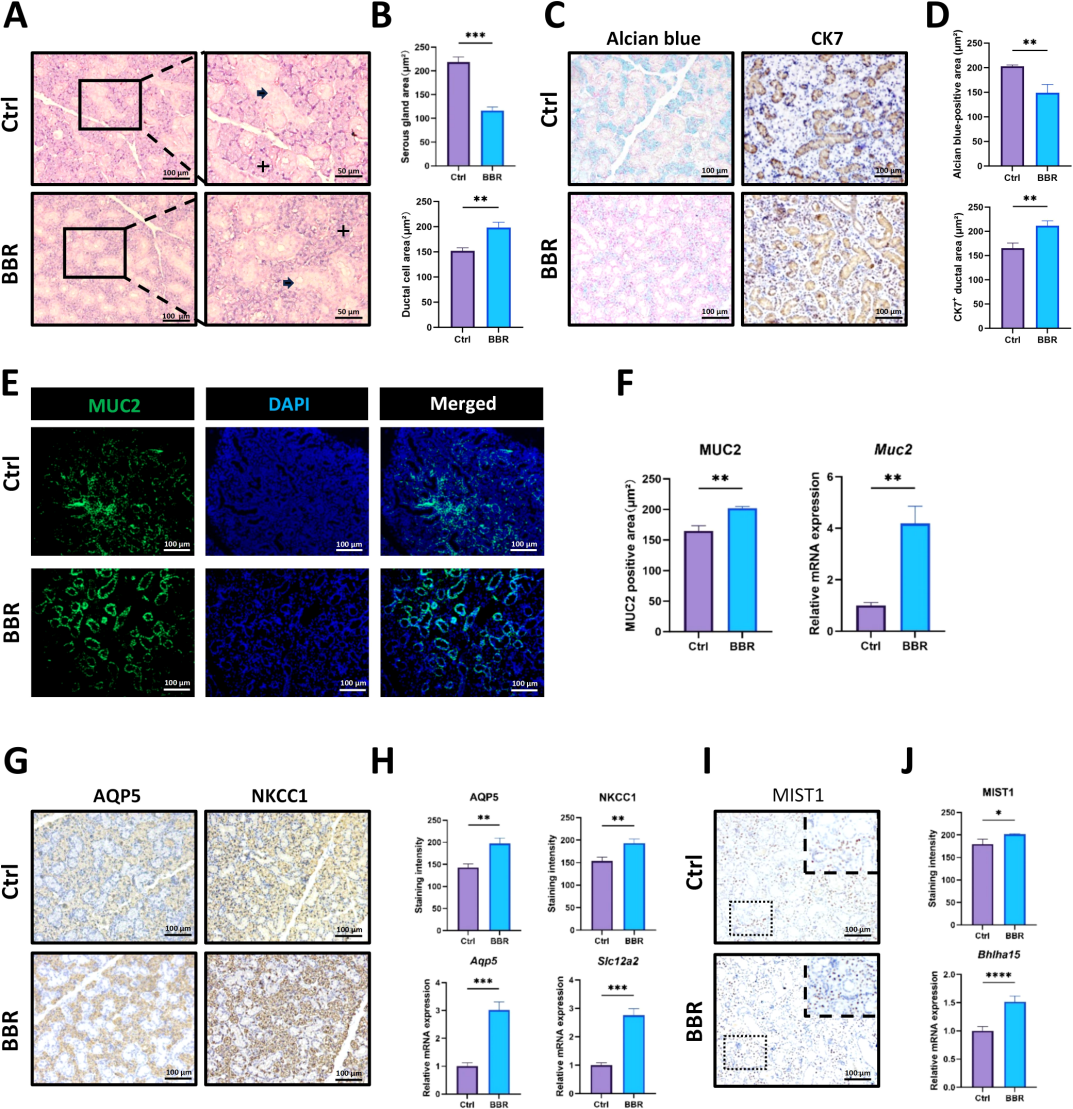
BBR significantly changes the histological characteristics of SMG tissues related to saliva secretion. **(A)** Representative H&E staining images of SMG tissues. The cross indicates the serous acinar gland, and the arrow indicates ducts. **(B)** Quantitative analysis of the ratios of serous gland and duct areas to the total tissue area (n=3 per group). **(C)** Representative images of Alcian blue staining and CK7 immunohistochemical (IHC) staining of SMG tissues from the control group and BBR group (Bar= 100 μm). **(D)** Quantitative comparison of Alcian blue-positive acinar area and CK7-positive ductal area in SMG tissues (n=3 per group). **(E)** Immunofluorescence (IF) staining for MUC2 to visualize ducts in the SMG of different groups (Bar= 100 μm). **(F)** Quantitative analysis of MUC2-positive ducts and mRNA expression levels of *Muc2* gene in each group (n=3 per group). **(G)** Representative IHC images of AQP5 and NKCC1 in the control and BBR groups (Bar= 100 μm). **(H)** Quantitative analysis of the staining intensity of AQP5 and NKCC1 and qPCR results of their gene expression levels (n=3 per group). **(I)** Representative images of IHC against MIST1 (Bar= 100 μm). **(J)** BBR treatment increased the intensity of MIST1 staining and mRNA expression of *Bhlha15* gene (n=3 per group). *P < 0.05, **P < 0.01, ***P < 0.001, ****P < 0.0001.

On the basis of the above-mentioned results, we further investigated how BBR influences the histology of SMG tissues and explored the potential reasons for increased saliva secretion and decreased SMG size. In the H&E images, staining revealed that the serous acinar cells were in clusters (indicated by cross) and were more hematoxylin-trophic and that mucus ductal cells (demonstrated by arrow) were stained by hematoxylin to a lesser degree (**Figure 2A**). BBR treatment significantly decreased the area of serous acinar cells but caused an expansion of the mucus ducts (**Figure 2B**). Alcian blue is usually used to stain acidic mucus in secretory cells, such as goblet cells in the gastrointestinal tract. In this study, we observed that it could specifically stain serous acinar cells in the SMG and that the area of Alcian blue-positive acinar cells was significantly decreased after the administration of BBR. Moreover, we stained cytokeratin 7 (CK7) to identify mucus-secreting ductal cells. It was observed that BBR caused an increase in these cells (**Figures 2C, D**), and BBR decreased the acini/ducts ratio (**Supplementary Figure S1**). We also stained MUC2 to visualize ductal cells and found more ducts in BBR-treated SMG, which was also confirmed by qRT-PCR (**Figures 2E, F**). Additionally, we analyzed the expression of AQP5 and NKCC1, which are two important saliva secretion-related functional proteins in the SMG. Our results revealed that, in BBR-treated mice, the staining intensity of AQP5 and NKCC1 in serous acinar cells was significantly increased, and the mRNA expression levels of *Aqp5* and *Slc12a2* were also greatly increased in response to BBR injection (**Figures 2G, H**). MIST1 has been identified as an important transcription factor that regulates the maturation and secretion of acinar cells, such as gastric chief cells, pancreatic acinar cells, Paneth cells, and salivary acinar cells (5). Therefore, we further examined its expression in BBR-treated SMG. We found that both IHC staining for MIST1 and mRNA levels of *Bhlha15* were significantly increased in acinar cells after BBR treatment (**Figures 2I, J**), which might lead to increased saliva secretion. Additionally, the western blot results showed similar changes to the staining images (**Supplementary Figure S2**). These data indicate that BBR can induce dramatic histological changes in SMG tissues *in vivo*, especially in acinar cells of healthy mice.

### 3.3 BBR influences the balance between proliferation and apoptosis in the SMG

**Figure 3:**
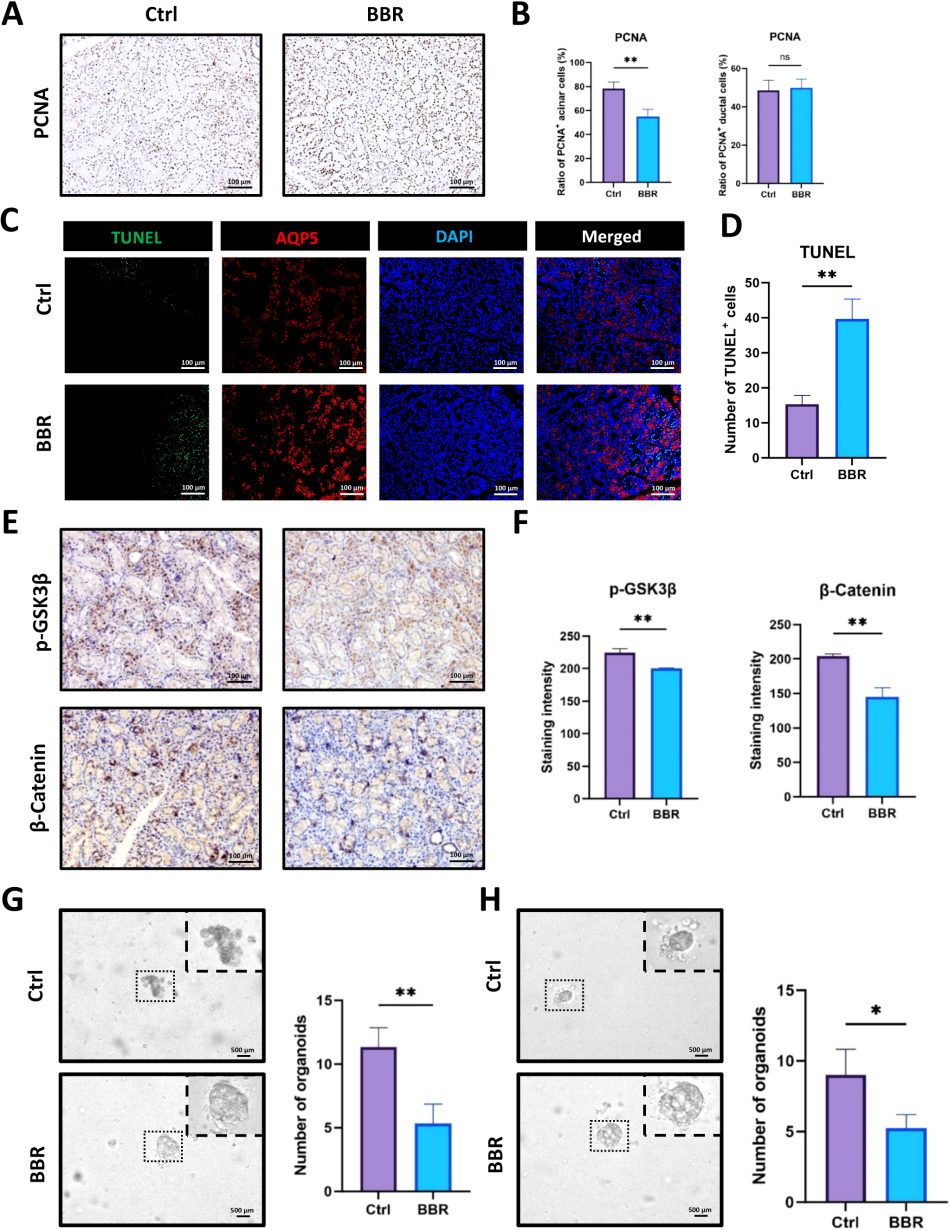
BBR affects proliferation, apoptosis and the Wnt/β-Catenin signaling pathway in the SMG tissues of healthy mice. **(A)** Representative IHC images of PCNA staining in SMG from control and BBR-treated mice (Bar= 100 μm). **(B)** Quantification of PCNA-positive acinar and ductal cells. **(C)** Representative staining images of AQP5(red) and TUNEL(green) at day 1 after BBR treatment (Bar= 100 μm). **(D)** Quantitative analysis of TUNEL positive cells per field (n=3 per group). **(E)** Representative IHC images for p-GSK3β and β-Catenin in the two groups (Bar= 100 μm). **(F)** Quantitative analysis for the staining intensity of p-GSK3β and β-Catenin between the control group and BBR-treated group (n=3 each group). **(G)** Representative images of SMG organoids cultured from healthy control and BBR-treated mice and quantitative comparison of the number of SMG organoids (Bar= 500 μm). **(H)** Representative images of SMG organoids cultured without or with BBR in the medium *in vitro* and quantitative comparison of the number of SMG organoids (Bar= 500 μm). *P < 0.05, **P < 0.01, *ns*, not significant.

Since BBR significantly changed the histology of SMG tissues, we wondered how BBR induced such changes. As the balance between cellular proliferation and apoptosis controls the homeostasis of different tissues, we firstly examined the differential expression of PCNA and TUNEL in control and BBR-treated SMG tissues. IHC staining for PCNA revealed that the administration of BBR greatly decreased the amount of PCNA^+^ cells, especially in serous acinar cells (**Figures 3A, B**). Moreover, we examined the level of apoptosis within SMG after BBR treatment using TUNEL assay. It was demonstrated that BBR treatment increased the number of TUNEL^+^ apoptotic cells at the early stage (**Figures 3C, D**). Wnt/β-Catenin is the most important signaling pathway that governs cell proliferation. Therefore, we examined the expression levels of GSK-3β and β-Catenin in SMG. Using IHC staining, we observed that p-GSK-3β was expressed mainly in the acinar cells of healthy SMG but was significantly decreased on day 7 after BBR treatment. Moreover, the staining level of β-Catenin was also decreased in ductal cells (**Figures 3E, F**), and the decreased protein level of β-Catenin was also proven by Western blot (**Supplementary Figure S2**). To validate these results, we further cultured SMG organoids from healthy and BBR-treated mice. The organoids derived from healthy SMG tissues grew more vigorously than BBR-treated SMG organoids did and seemed to have more buds and lobes. BBR-treated organoids could only grow in thinner spheres. Moreover, healthy SMG tissues formed more organoids with better morphological characteristics than BBR-treated SMG tissues did (**Figure 3G**). We cultured SMG organoids from healthy mice and then treated them with BBR *in vitro*, and the same results were reproduced in SMG organoids (**Supplementary Figure S3A**, **Figure 3H**). Since LiCl is a frequently used activator of Wnt pathway, we tested the combination of BBR and LiCl. Interestingly, the combination increased the number of SMG organoids compared to BBR group (**Supplementary Figure S3B**). These results demonstrate BBR is capable of affecting the balance between proliferation and apoptosis in SMG tissues under physiological conditions via interruption of the Wnt/β-Catenin signaling pathway.

### 3.4 BBR treatment relieves HNR-induced xerostomia in mice

**Figure 4:**
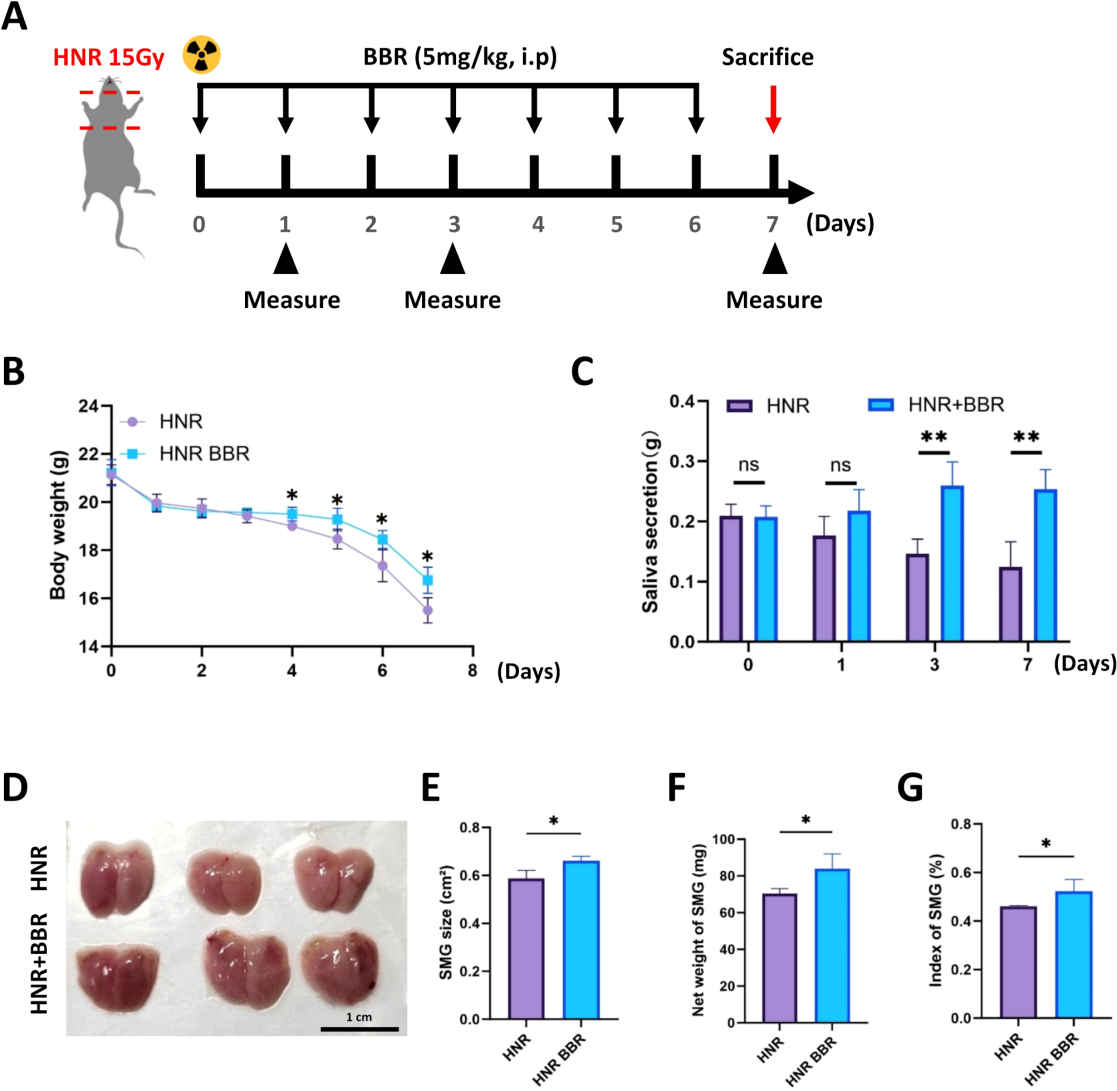
BBR greatly mitigates HNR-induced xerostomia in mice. **(A)** Schematic diagram of the experimental design. **(B)** BBR attenuated the loss of body weight after HNR-induced injury (n=4 per group). **(C)** BBR treatment significantly alleviated the reduction in saliva secretion after HNR-induced xerostomia (n=4 per group). **(D)** The appearance of SMG in the HNR group and BBR group (n=3 each group, Bar=1 cm). **(E)** Surface area, **(F)** net weight, and **(G)** normalized index of SMG in control and BBR-treated mice (n=4 per group). *P < 0.05, **P < 0.01, *ns*, not significant.

Given the effects of BBR on healthy mice that we observed, we aimed to examine whether BBR could also be beneficial for the recovery of HNR-induced SMG injury. Therefore, we established an HNR model to investigate this hypothesis. HNR and BBR treatment were performed according to the indicated protocol (**Figure 4A**). Specifically, BBR was administered by intraperitoneal injection at a dose of 5 mg/kg for 6 consecutive days. First, we observed the body weights of the mice in the different groups. Both the HNR group and the HNR+BBR group presented a decrease in mean body weight, but BBR-treated mice lost weight more slowly (**Figure 4B**). Owing to HNR-induced salivary injury, there was a significant decrease in saliva secretion in HNR-treated mice. However, BBR treatment promoted the saliva secretion at days 3 and 7 (**Figure 4C**). The gross morphological appearance of the SMG in these two groups was shown in **Figure 4D**. Furthermore, the size, net weights and normalized indices of the SMG were also significantly greater in the BBR-treated mice than in the HNR-injured mice (**Figures 4E–G**). Collectively, these data clearly show that HNR significantly reduces saliva secretion and SMG volume, but BBR could clearly mitigates the radiation induced injury in SMG tissues by increasing the gland weight, expanding the size of acini and ducts, etc.

### 3.5 BBR preserves the morphology and function of SMG tissues after HNR-induced injury

**Figure 5:**
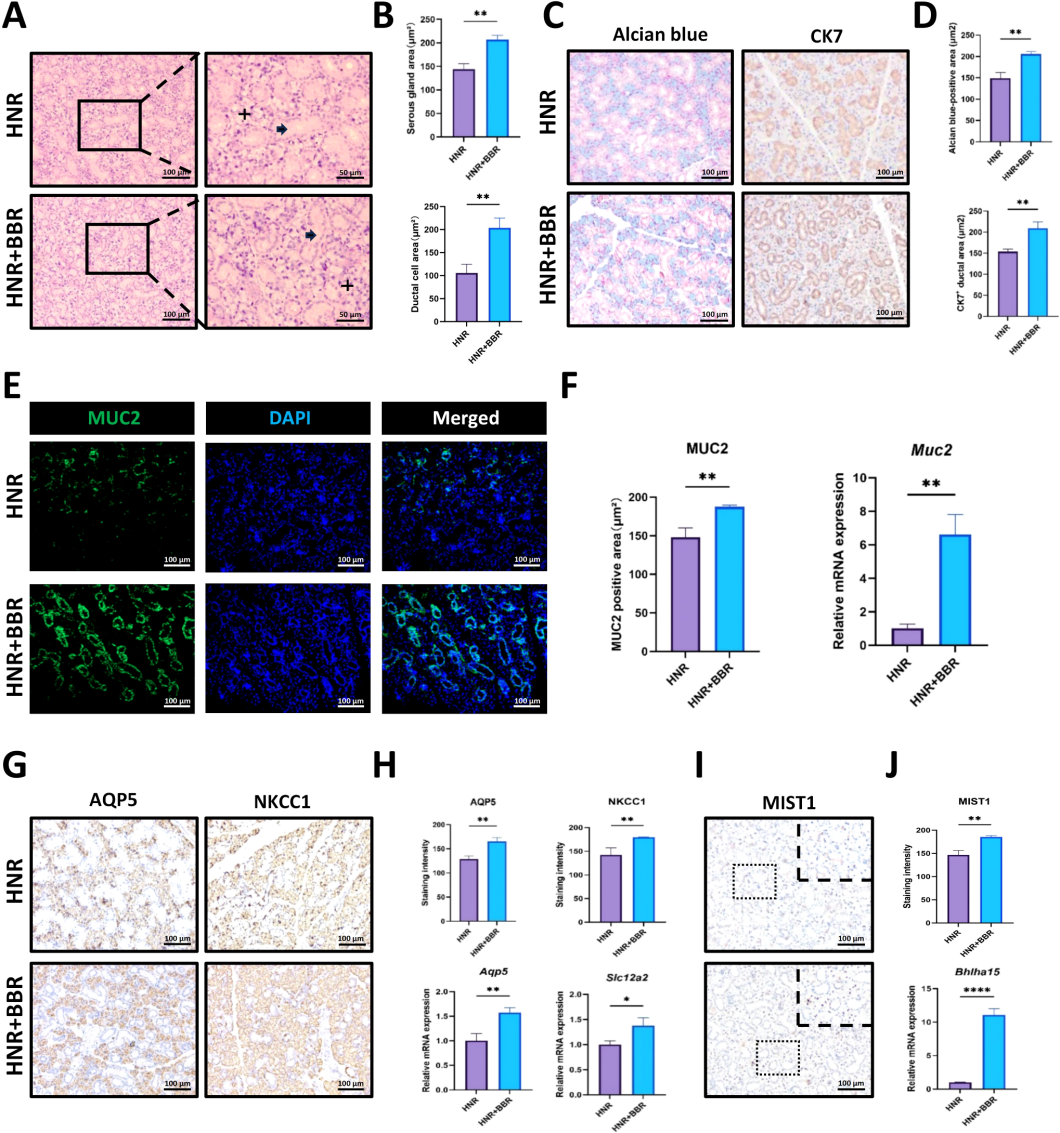
BBR alleviates HNR-induced morphological and functional damage to SMG tissues. **(A)** Representative H&E images of SMG tissues from each group after HNR-induced injury. The arrow indicates ducts, and the cross indicates the acinar gland. **(B)** Quantitative analysis comparison of the ratio of acinar and ductal areas in total SMG tissues (n=3 per group). **(C)** Images of Alcian blue and CK7 staining in the HNR group and BBR group (Bar= 100 μm). **(D)** Quantitative comparison of Alcian blue-positive acinar glands and CK7-positive ducts in each group (n=3 per group). **(E)** Representative IF staining images of MUC2 (Bar= 100 μm). **(F)** BBR treatment significantly increased the staining intensity of MUC2 and the mRNA expression of *Muc2* gene (n=3 per group). **(G)** Representative IHC images of AQP5 and NKCC1 revealed more positive cells in BBR-treated SMG (Bar= 100 μm). **(H)** Quantitative analysis of the IHC intensity of AQP5 and NKCC1 and the mRNA expression levels of the *Aqp5* and *Slc12a2* genes (n=3 each group). **(I)** IHC staining of MIST1 in the HNR group and BBR group. **(J)** Quantitative analysis of the intensity of MIST1 staining and the mRNA expression of the *Bhlha15* gene (n=3 per group). *P < 0.05, **P < 0.01, ****P < 0.0001.

We further examined how BBR influenced the histopathology of the SMG following HNR-induced injury. H&E staining revealed profound shrinkage of acinar cells and mucus ducts in HNR-treated mice. However, the administration of BBR preserved mature acinar cells (indicated by cross) and expanded the ductal area (shown by arrow), indicating attenuated histological and structural disruption of the SMG, along with a markedly reduced severity of edema (**Figures 5A, B**). Simultaneously, we also performed Alcian blue and CK7 staining to visualize acinar cells and duct cells, respectively. There were more plump Alcian blue-positive acinar cells in BBR-treated HNR mice than in HNR control mice, and the area of CK7-positive ducts in BBR-treated HNR mice was significantly greater than that in control HNR mice (**Figures 5C, D**). Additionally, we used MUC2 to identify ductal cells in the SMG. We observed more MUC2-positive ductal cells in the mice that received BBR treatment than in the control mice, and they also had higher mRNA levels of the *Muc2* gene (**Figures 5E, F**). In addition to morphological analysis, we explored the expression patterns of AQP5, NKCC1 and MIST1, which are important regulators of saliva secretion, in acinar cells. The staining images and qPCR results revealed that IHC staining for both AQP5 and NKCC1 was much stronger in BBR-treated acinar cells with a plump morphology than in control acinar cells and that their mRNA levels were also significantly greater than those in mice with HNR-induced injury (**Figures 5G, H**). MIST1 governs the formation and maturation of secretory granules in glandular cells. HNR injury led to the loss of this protein and mRNA. However, BBR injection greatly alleviated this decrease, which helped preserve saliva secretion function (**Figures 5I, J**). And the protein levels of AQP5 and MIST1 were clearly increased as shown by Western blot (**Supplementary Figure S4**). Therefore, our results indicate that BBR enhances salivary preservation against HNR-induced injury in SMG.

### 3.6 BBR alleviates radiation-induced hypoproliferation and inflammation in HNR mice

**Figure 6:**
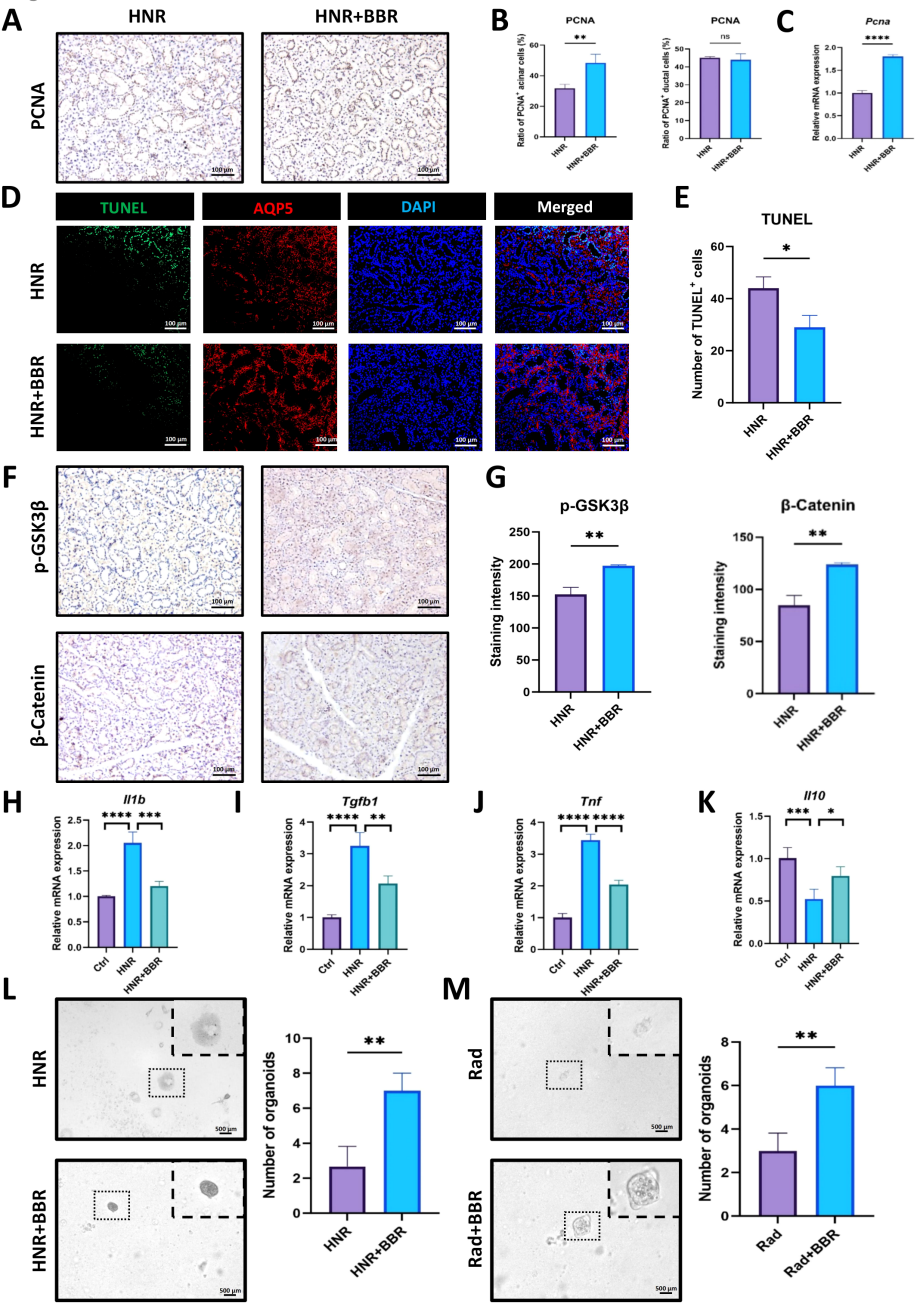
BBR regulates cell proliferation, apoptosis, and inflammation in response to HNR-induced SMG injury. **(A)** BBR increased the number of PCNA-positive cells within SMG tissues after HNR injury (Bar= 100 μm). **(B)** Quantitative analysis of PCNA-positive acinar and ductal cells (n=3 per group). **(C)** Comparison of the mRNA expression level of the *Pcna* gene by qPCR assay (n=3 per group). **(D)** Representative images of AQP5 (red) and TUNEL (green) staining of SMG in different groups (Bar= 100 μm). **(E)** Statistical analysis for the number of TUNEL^+^ cells after HNR injury (n=3 per group). **(F)** Representative IHC images revealed that higher positive signals of p-GSK3β and β-Catenin in BBR-treated HNR mice (Bar= 100 μm). **(G)** Comparison of the staining intensity of p-GSK3β and β-Catenin between the two groups (n=3 each group). **(H-K)** qRT-PCR results for the expression of inflammatory genes in the two groups, including *Il-1b*, *Tgfb1*, *Tnf*, and *Il-10* in SMG tissues of HNR- and BBR-treated HNR- groups (n=3 per group). **(L)** Representative images of SMG organoids derived from HNR- and BBR-treated HNR- mice and statistical analysis of the number of SMG organoids in the two groups. **(M)** Representative images of SMG organoids at day 7 after a single dose of 5 Gy radiation without or with BBR treatment *in vitro* and statistical analysis of the number of SMG organoids in the two groups. *P < 0.05, **P < 0.01, ***P < 0.001, ****P < 0.0001, *ns*, not significant.

**Figure 7:**
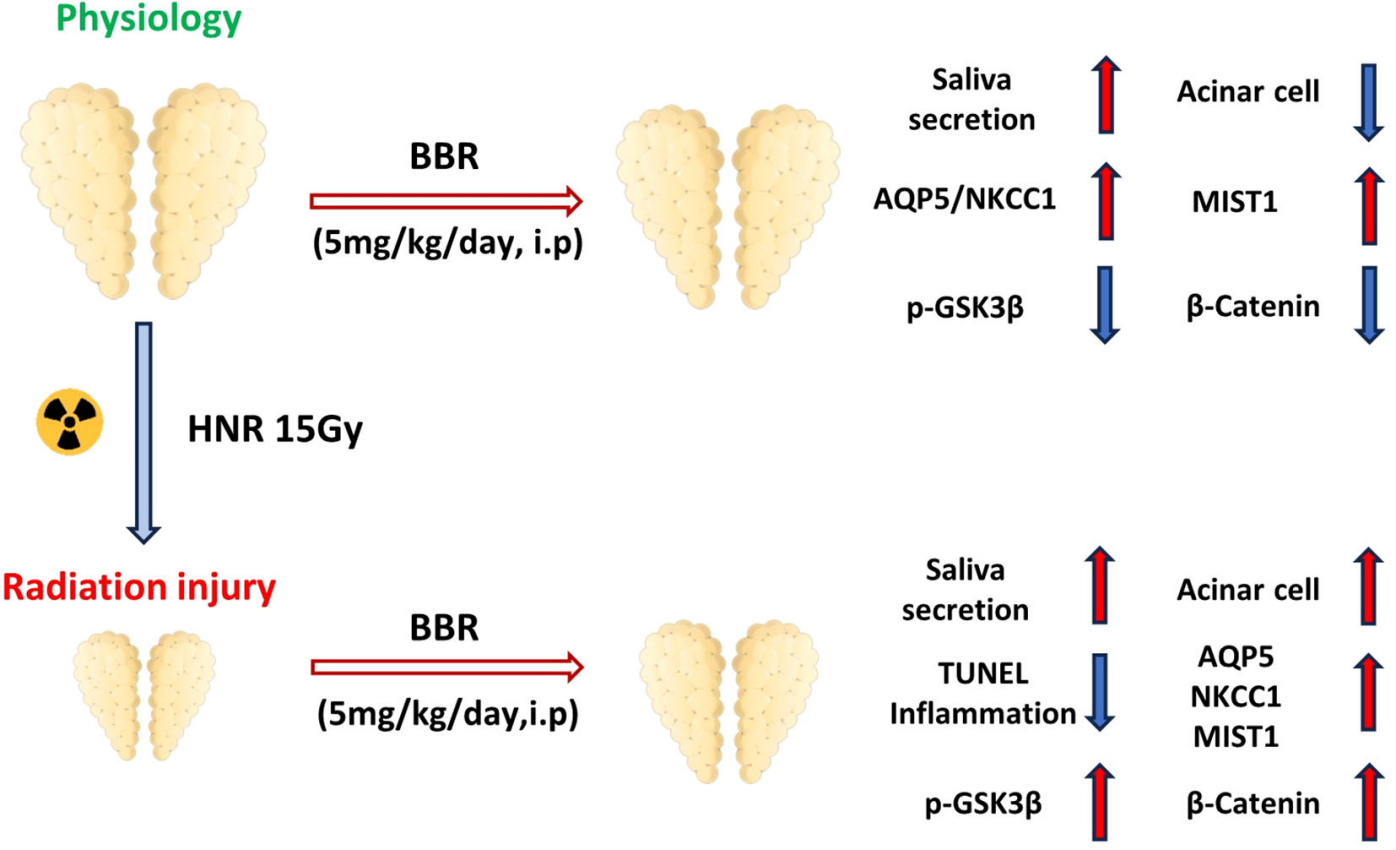
Schematic illustration of how BBR enhanced the saliva secretion under in homeostasis and after radiation injury.

Owing to the previously described effects, we also investigated whether BBR could modulate proliferative dysfunction, apoptosis, and the inflammatory response in the SMG of HNR-injured mice. First, we performed IHC staining for PCNA. The mice in the BBR group presented more PCNA-positive signals, especially in acinar cells, and the results of quantitative analysis and qPCR confirmed these results (**Figures 6A–C**), which explained why mice in BBR-treated HNR group had a bigger SMG size and more saliva secretion. Epithelial cells in SMG cells undergo apoptosis due to radiation exposure. TUNEL^+^ cells were significantly reduced in BBR-treated mice at day 1 after HNR injury (**Figures 6D, E**), indicating that apoptosis was alleviated in SMG. As described previously, the Wnt/β-Catenin pathway is crucial for maintaining the function of saliva secretion in SMG tissues. Interestingly, BBR-treated mice presented more p-GSK3β staining signals in acinar cells after HNR injury, and the β-Catenin level in the BBR group was also greater than that in the HNR group (**Figures 6F, G**, **Supplementary Figure S4**). Moreover, radiation causes damage to tissues and is accompanied by the activation of inflammatory factors. The infiltration of inflammatory cytokines impairs the function of the salivary gland (24). qPCR revealed that ionizing exposure increased the mRNA levels of proinflammatory cytokines (*Il1b*, *Tgfb1*, and *Tnf*) and inhibited the expression of *Il10*, which is an anti-inflammatory factor, but BBR significantly reversed these changes (**Figures 6H–K**). Additionally, we isolated SMG cells from HNR- or BBR-treated SMG tissues to culture organoids. BBR significantly improved the formation and growth of SMG organoids, indicating increased viability of SMG cells in BBR-treated HNR mice (**Figure 6L**). We also irradiated healthy SMG organoids and treated them with BBR *in vitro*, and we observed the similar results (**Figure 6M**). When XAV939, the inhibitor of Wnt signaling, was incubated with irradiated SMG organoids, the promoting effects of BBR could be decreased (**Supplementary Figure S5**). Taken together, these data indicate that BBR treatment greatly improves cell proliferation, inhibits apoptosis, and alleviates inflammation in SMG tissues after HNR-induced xerostomia (**Figure 7**).

## 4 Discussion

Radiotherapy is the cornerstone therapeutic intervention for head and neck malignancies. However, while radiotherapy increases local and regional control of cancers and improves the overall survival of these patients, it consistently leads to predictable treatment-related adverse effects. Among all the clinical symptoms among patients receiving head and neck radiotherapy (HNR), salivary hypofunction is the predominant acute and chronic sequela and is characterized by irreversible glandular dysfunction and progressive xerostomia (7). Early HNR complications include mucositis, swallowing difficulty, vocal alterations, and clinically significant xerostomia, which profoundly compromise the quality of life of patients. Current therapeutic strategies include anti-inflammatory pharmacotherapy (25), mesenchymal stem cell transplantation, and surgical gland preservation techniques (26–28). Unfortunately, all these approaches remain investigational without a consensus on the optimal treatment protocols, and their clinical translation is hindered by inconsistent efficacy profiles and unresolved safety concerns. Therefore, novel, cost-effective pharmacological interventions targeting xerostomia pathogenesis are urgently needed.

Serous acinar glands are the major site for the production of saliva, so their healthy morphology and function are essential for the treatment of xerostomia. Acinar cells express high levels of AQP5, NKCC1, and MIST1 to support their secretion function and the maturation of secretory granules. AQP5 is a water channel protein that mediates water transport across the plasma membrane along its osmotic gradient (28). NKCC1, which is also known as solute carrier family 12 member 2 (SLC12A2), is required for the electroneutral transportation of chloride, potassium and/or sodium ions across the membrane (29). MIST1 is also known as basic helix-loop-helix family member a15 (bHLHA15) and has been shown to regulate the formation and maturation of granule particles in pancreatic acinar cells (5), Paneth cells (30), and gastric chief cells (31). In this study, we examined the histological staining and mRNA expression levels of these molecules to assess the secretory functions of SMG in mice.

In traditional Chinese medicine, the herb Coptis has been used in clinical practice for thousand years. BBR is the most useful bioactive component of Coptis and is widely prescribed to treat many clinical diseases, especially gastrointestinal disorders. Emerging evidence suggests that BBR exerts its pharmacological effects by modulating the inflammatory response and antioxidant efficacy in chronic disease models (32, 33). Notably, BBR can inhibit apoptosis and has demonstrated cytoprotective effects across multiple pathological contexts (34, 35). BBR promotes proliferation to protect the intestinal mucosa (21). Mechanistically, BBR increases tissue regeneration by activating the Wnt/β-Catenin pathway (36). Recently, we identified its positive function in modulating intestinal stem cells and their microenvironment (13). Despite these advancements, the therapeutic potential and mechanistic basis of the role of BBR in xerostomia pathogenesis have not been carefully explored. Therefore, given these established correlations, in this study, we systemically investigated whether and how BBR regulated the secretory function of the salivary gland in mice.

First, we discovered that BBR promoted the saliva secretion of SMG in healthy mice (**Figure 1C**) and the volume of the SMG was decreased. H&E and Alcian blue staining also revealed the obvious shrinkage of acinar cells (**Figures 2A, C**). This observation suggests that the functional improvement is not attributable to glandular hypertrophy but may instead involve a shift in the composition of the glandular tissue. We propose a model of functional compensation and tissue remodeling. Critically, our histological analysis revealed a significant decrease in the acini/ducts ratio in BBR-treated glands (**Supplementary Figure S1**), this indicates that the functional improvement is not solely driven by an expansion of the secretory acinar compartment. We observed a BBR-induced expansion of the ductal cell population, as the ductal network serves as the essential conduit for saliva transport, its strategic enlargement likely enhances the gland's excretory capacity, ensuring efficient delivery of saliva from the secretory units to the oral cavity. This structural optimization is further amplified at the molecular level by a marked upregulation of key secretory proteins, AQP5 and NKCC1, within the acinar compartment. We propose that under physiological conditions, BBR remodels the salivary gland into a more efficient organ through functional refinement rather than tissue growth. Despite the reduction in gland mass and volume, this outcome is fully compensated by the combined benefits of an optimized tissue composition and enhanced secretory capacity per cell. Our data most strongly support that the resolution of this paradox lies in a qualitative enhancement of the gland's functional infrastructure. Subsequent IHC staining confirmed the loss of PCNA^+^ proliferative acinar cells (**Figure 3A**), which should be the cellular basis for the decreased volume of the SMG. Since SMG cells have been shown to have a high proliferative potential (37), these data provide evidence that BBR suppresses proliferation of them.

Wnt/β-Catenin is the most important signaling pathway responsible for cell proliferation. Therefore, we observed the significant decreases in these two proteins in acinar cells after HNR injury. Stem cells in SMG tissues have been reported to located adjacent to the ductal cells (37). Interestingly, the distribution of β-Catenin-positive cells followed the same pattern, which may imply diminished stem cell proliferation (**Figures 3E, F**), indicating that BBR caused the hypofunction of the Wnt/β-Catenin pathway in the SMG of healthy mice. Intriguingly, IHC staining for AQP5, NKCC1, and MIST1 revealed an obvious increase in both their protein intensities and levels of mRNA expression (**Figures 2G–J**). These enhancement of secretory protein combined with an expansion of the ductal compartment that likely improves salivary conduit function compensates for the observed reduction in acinar cell area, which might explain why BBR could enhance saliva secretion during homeostasis. Moreover, consistent with the *in vivo* results, BBR treatment resulted in fewer and less densely packed organoids, regardless of whether the treatment was administered *in vivo* prior to cell isolation or *in vitro* directly to the organoid culture. (**Figures 3G, H**), and BBR-mediated suppressive effect was antagonized by the Wnt agonist LiCl in the organoid model (**Supplementary Figure S3B**).

In the context of HNR-induced injury, we observed a progressive decrease in body weight and gradually decreased saliva secretion. The size and net weight were wholly lower than those of healthy SMG. These results are consistent with those of previous studies (38, 39). Notably, the administration of BBR significantly alleviated the above-mentioned deficiency of SMG (**Figures 4B–G**). Conversely, BBR restored salivary function in HNR-injured glands. We interpret this not as a failure, but as evidence of a distinct reparative strategy. Following severe injury, the paramount task is rapid tissue regeneration. We propose that BBR initially promotes a robust, generalized proliferation of all progenitor or ductal cells to rebuild the glandular infrastructure. The functional enhancement was accompanied by increased expression of AQP5, NKCC1 and MIST1 (**Figures 5G–J**), which contributed to preservation of the original morphology and function of acinar cells in SMG. SMG stem cells are located mainly in ductal cells (37), and saliva secreted by the serous gland is partly delivered by intercellular canaliculi between ductal cells (40). We found that BBR increased the number of PCNA-positive acinar cells and the mRNA expression (**Figures 6A–C**). And the activity of Wnt/β-Catenin signaling pathway was partially maintained. Therefore, we propose that BBR increases the proliferation of acinar cells, and at the same time BBR caused the expansion of CK7^+^ ducts tend to facilitate salivary efflux. Moreover, our results demonstrated that BBR treatment significantly inhibited the severity of the inflammatory response, which was indicated by the altered expression of *Il1b*, *Tgfb1*, *Tnf*, and *Il10* (**Figures 6H–K**). Given that our study found BBR upregulates AQP5 and NKCC1 expression in the SMG and alleviates tissue injury, and considering that BBR has been demonstrated to mitigate inflammation in the intestinal mucosa by suppressing pro-inflammatory cytokines and modulating local immune homeostasis (19), it is plausible that it operates via a similar immunomodulatory mechanism in the SMG. BBR promoted the growth and morphological recovery of organoids under HNR-injured conditions. This result aligns with our *in vivo* data and reinforces the conclusion that BBR's function is not monolithic but adapts to the pathological state of the tissue.

Despite these exciting results, the present study also has several limitations. First, BBR reportedly modulates the metabolism of the gut microbiota (41). Increasing evidence has revealed a possible connection between the gut microbiota and organ health outside the gastrointestinal tract. We are also exploring whether the gut flora and its metabolites are helpful for the pathogenesis and treatment of xerostomia. As such, further research is needed to elucidate the effects of BBR on the gut microbiota to alleviate the severity of radiation-induced xerostomia. Second, although we found that BBR can regulate the Wnt/β-Catenin pathway in SMG, the exact molecular interaction between BBR and signaling-related proteins remains unclear. Although our functional data firmly establish that BBR regulates the Wnt/β-Catenin pathway, the exact molecular interaction, such as direct binding to GSK3β or the stabilization of β-Catenin, remains a subject for future investigation. The observed antagonism of XAV939 and LiCl's effects strongly suggests that BBR's action converges on the Wnt signaling. Finally, the present study clearly revealed that BBR causes apparent shrinkage of healthy SMG, with atrophy of acinar glands, although it preserves the size and morphology of SMG tissues after HNR-induced injury. While we have proposed a mechanistic hypothesis for this phenomenon in the present study, the opposite biological effects of BBR on healthy and radiation-damaged SMG should be further investigated in the future.

In conclusion, our study provides the first evidence that BBR can directly protect salivary gland function against injury, uncovering a novel, dual role in bidirectionally modulating the Wnt/β-Catenin pathway that inhibiting it in healthy states and activating it in damaged states. Furthermore, we resolve the paradox of enhanced secretion from a smaller gland by demonstrating that BBR induces functional tissue remodeling. And after radiation-injured, it initiates a comprehensive repair program that restores secretory function despite an initial phase of structural reorganization. This study reveals that BBR is an inexpensive, orally available drug with a well-established safety profile. Our findings position it as a highly promising, clinically translatable therapeutic candidate for preventing and treating radiation-induced xerostomia.

## Data Availability

The original contributions presented in the study are included in the article/**Supplementary Material**. Further inquiries can be directed to the corresponding authors.
