# Supplementary material for: Berberine augments the secretory function of salivary gland in homeostasis and after radiation exposure
Source: Front Immunol. 2026 Jan 12;16:1685137. doi: 10.3389/fimmu.2025.1685137 (PMC12832237; doi:10.3389/fimmu.2025.1685137)
Supplement: Supplementary file 1 [file DataSheet1.docx]

**Supplementary materials**


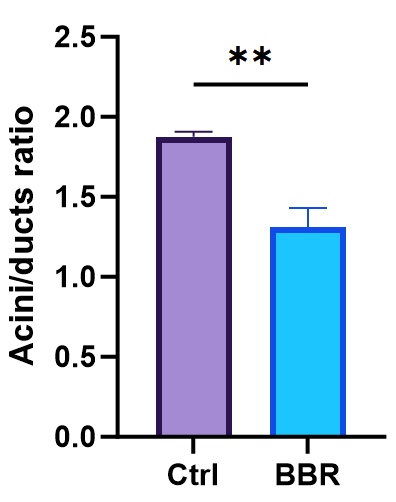


**Figure S1. BBR significantly decreases the acinar-to-ductal ratio of SMG in physiology.** Quantification results for the acini/ducts ratio of SMG in physiological condition with or without BBR treatment. ** *P*< 0.01.


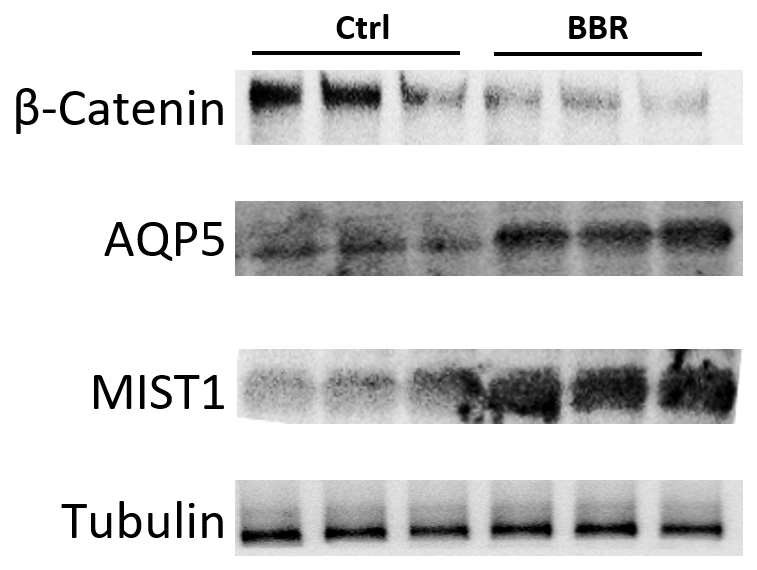


**Figure S2. BBR treatment declines the protein expression of β-Catenin and increases the secretory proteins in physiological conditions.** Western blot images of β-Catenin, AQP5, and MIST1 protein expression in SMG of control and BBR-treated mice under physiological conditions. Tubulin was used as loading control (n=3 per group).


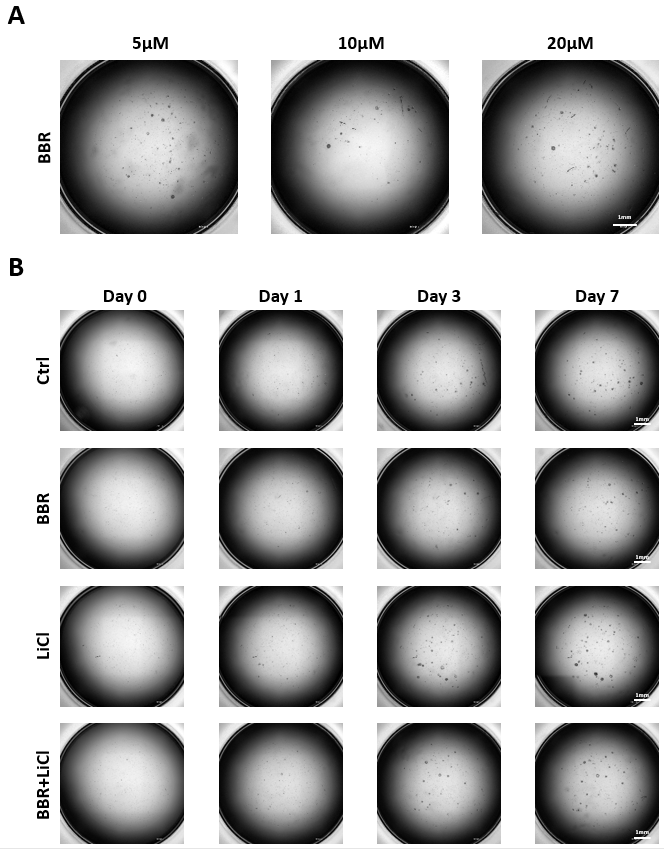


**Figure S3. BBR regulates the growth of SMG organoids in physiology by Wnt/β-Catenin signaling.** (A) Optimization for the doses of BBR before treating SMG organoids (Bar=1mm). (B) Freshly isolated SMG cells were immediately treated by BBR, LiCl or their combination. The images of SMG organoids were captured at Day 0, Day 1, Day 3, and Day 7 (Bar=1mm).


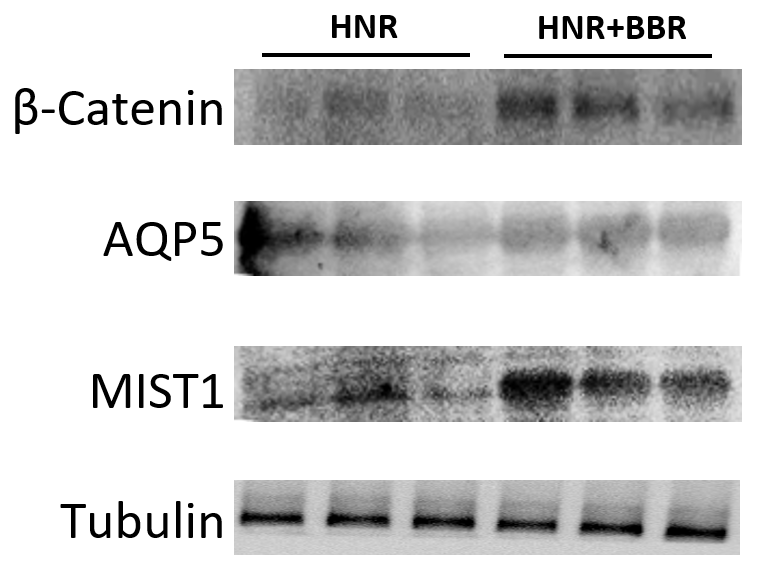


**Figure S4. BBR treatment increases the protein expression of β-Catenin and secretory proteins in SMG tissues after HNR injury.** Western blot results of β-Catenin, AQP5, and MIST1 protein expression in SMG tissues of control and BBR-treated mice at Day 7 after HNR. Tubulin was adopted as the loading control (n=3 per group).


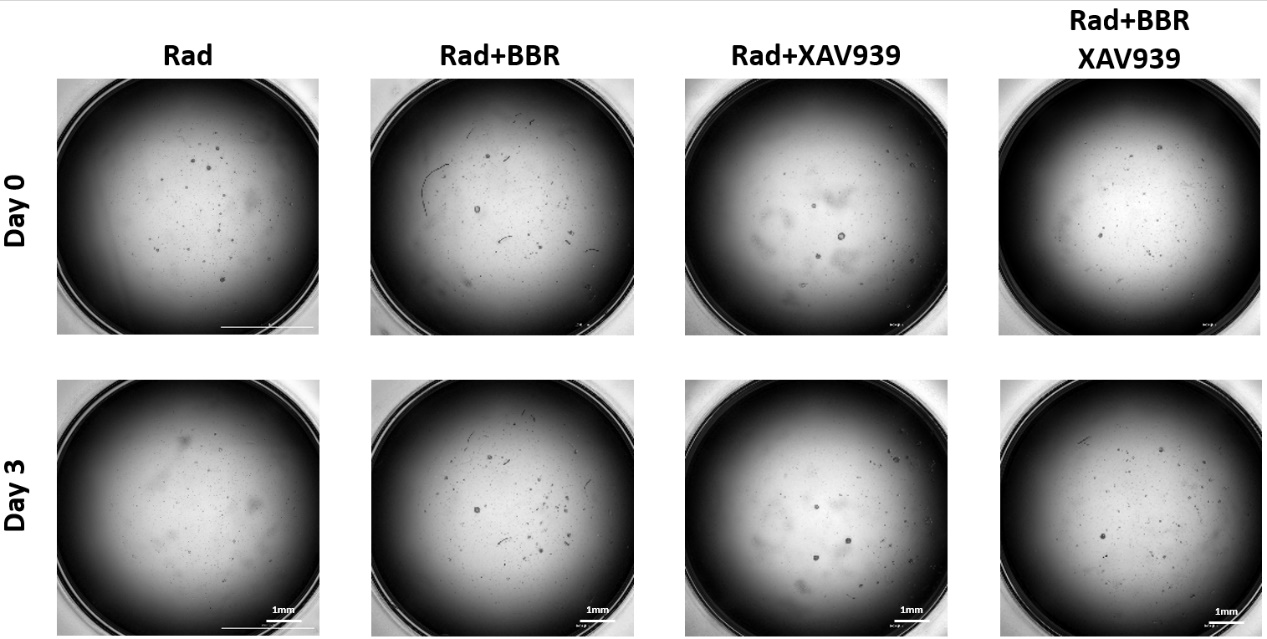


**Figure S5. BBR requires the activation of Wnt signaling to strengthen the repairing of SMG organoids after 6 Gy radiation injury.** SMG organoids were cultured to the mature status, and then received an irradiation of 6 Gy exposure. BBR, XAV939 and the combination were respectively administered to treat SMG organoids for 3 days (Bar=1mm).

**Table S1. Information of Antibodies used in this study**

| **Antibody** | **Provider** | **Catalog** | **Host** | **Dilution** |
| --- | --- | --- | --- | --- |
| AQP5 | Abcam | ab92320 | Rabbit | Staining assay: 1:400  Western blot: 1:2000 |
| β-Catenin | BD | 610153 | Mouse | Staining assay: 1:200  Western blot: 1:2500 |
| CK7 | Proteintech | 17513-1-AP | Rabbit | Staining assay: 1:400 |
| p-GSK3β | Proteintech | 67558-1-Ig | Mouse | Staining assay: 1:200 |
| MUC2 | Proteintech | 27675-1-AP | Rabbit | Staining assay: 1:400 |
| NKCC1 | Proteintech | 13884-1-AP | Rabbit | Staining assay: 1:200 |
| MIST1 | Proteintech | 68092-1-Ig | Mouse | Staining assay: 1:250  Western blot: 1:4000 |
| PCNA | Abcam | ab92552 | Rabbit | Staining assay: 1:400 |
| Tubulin | Proteintech | 11224-1-AP | Mouse | Western blot: 1:5000 |

**Table S2. Primer sequences used for qRT-PCR assay**

| **Gene** | **Forward primer** | **Reverse primer** |
| --- | --- | --- |
| *Actb* | CTTCTTTGCAGCTCCTTCGTT | TTCTGACCCATTCCCACCA |
| *Aqp5* | AGAAGGAGGTGTGTTCAGTTGC | GCCAGAGTAATGGCCGGAT |
| *Bhlha15* | TCCTACGGGAGGCAGCAGT | TGTGTAGAGTAGCGTTGCAGG |
| *Il1b* | ACCTCACAAGCAGAGCACAA | TTGGCCGAGGACTAAGGAGT |
| *Il10* | TAAGGCTGGCCACACTTGAG | GTTTTCAGGGATGAAGCGGC |
| *Pcna* | TTGCACGTATATGCCGAGACC | GGTGAACAGGCTCATTCATCTCT |
| *Slc12a2* | TTCCGCGTGAACTTCGTGG | TTGGTGTGGGTGTCATAGTAGT |
| *Tgfb1* | GTCACTGGAGTTGTACGGCA | TCATGTCATGGATGGTGCCC |
| *Tnf* | ATGGCCTCCCTCTCATCAGT | TTTGCTACGACGTGGGCTAC |
